# Supplementary material for: Individual-based genetic analyses support asexual hydrochory dispersal in Zostera noltei
Source: PLoS One. 2018 Aug 16;13(8):e0199275. doi: 10.1371/journal.pone.0199275 (PMC6095491; doi:10.1371/journal.pone.0199275)
Supplement: S1 File — (DOCX) [file pone.0199275.s002.docx]

**S1, Supporting information 1**

Buga Berković, Nelson Coelho, Licínia Gouveia,^,^ Ester A. Serrão, Filipe Alberto

**Individual based genetic analyses support asexual hydrochory dispersal in Z*ostera noltei***

**Estimating clonal age using rhizome elongation rates and paleoceanographical sea-level change**

Seagrass clone longevity has been estimated assuming that the sampled spatial extent of a clone is the result of rhizome elongation alone. Using this approach the observed distance between sampled clonemates (samples belonging to the same multi-locus genotype, MLG) is simply divided by the distance horizontal rhizomes can elongate on average during one year [1,2]. We used a similar strategy, but iteratively corrected age estimates by combining sampling locations with spatial explicit sea-level data for the time the clone would have been initiated. Once clonal assignment was determined, we calculated the distance between each pair of clonemates belonging to the same genet. This “as the crow flies” distance, i.e., shortest distance between the two points on the ellipsoid, which does not consider possible land obstacles on the way, such as saltmarsh banks that never get submerged. The longest distance between a pair of clonemates of the same genet was used to define a minimum (i.e., sampled) span for that genet. For the purpose of the following methodological description, we shall refer to these points as A and B. We estimated the age for all clones sampled multiple times in the following way: First, using a conservative approach we assumed that the clone initiated growth at some middle point (O_0_) between A and B. Thus, an initial estimate of clone age (t_0_) was calculated by dividing the distance from O_0_ to A (half the clone’s span) by *Z. noltei* growth rate. We used a mean rate of rhizome elongation of 68 cm/year extracted from data published in a review [3], including studies from different areas and seasons. We chose to use species’ distribution wide data, instead of estimates for our study area. The rationale was that this is a better representation of growth rate variation through time, reflecting likely changes in habitat and climate. We note that the above method assumes asexual hydrochory to be impossible, because otherwise age estimation is unworkable. The above estimated clone’s age, was used as an initial estimate of how many years before the present the clone could have originated (t_0_) at the point O_0_. Using published paleoceanographic data on sea-level change in the Southern Portugal [4] and the current bathymetry, we mapped sea level changes over the past 20 000 years. We then verified if the origin point O_0_ was, at time t_0_, in suitable habitat (i.e. shallow coastal zone). If O_0_ was indeed in the coastal zone we assumed this to be the final age estimate. If on the contrary O_0_ was above sea level, we moved it to the closest coast line at that time before the present. Again, we placed this new point, O_1_, at an intermediate distance from A and B. Then we measured the distance to the points A and B and used this distance to update the age of the clone (t_1_). We repeated the above process, verifying if at the time t_1_, the point O_1_ would have been on the coast. The process was thus iterated until either reaching an origin point O_n_, which was at a certain time t_n_ before the present in the coastal zone, or until there was no more available data on sea level change for the region (Fig. S1).

**References**

1. Reusch TBH, Stam WT, Olsen JL. Size and estimated age of genets in eelgrass, Zostera marina, assessed with microsatellite markers. Mar Biol. 1999;133: 519–525.

2. Arnaud-Haond S, Duarte CM, Diaz-Almela E, Marbà N, Sintes T, Serrão EA. Implications of extreme life span in clonal organisms: millenary clones in meadows of the threatened seagrass Posidonia oceanica. PLoS One. Public Library of Science; 2012;7: e30454.

3. Marba N, Duarte CM. Rhizome elongation and seagrass clonal growth. 1998;174: 269–280.

4. Dias JMA, Boski T, Rodrigues A, Magalhães F. Coast line evolution in Portugal since the Last Glacial Maximum until present—a synthesis. Mar Geol. Elsevier; 2000;170: 177–186.
