# Supplementary material for: Individual-based genetic analyses support asexual hydrochory dispersal in Zostera noltei
Source: PLoS One. 2018 Aug 16;13(8):e0199275. doi: 10.1371/journal.pone.0199275 (PMC6095491; doi:10.1371/journal.pone.0199275)
Supplement: S2 File — (DOCX) [file pone.0199275.s003.docx]

**S2, Supporting information 2**

Buga Berković, Nelson Coelho, Licínia Gouveia,^,^ Ester A. Serrão, Filipe Alberto

**Individual based genetic analyses support asexual hydrochory dispersal in *Zostera noltei***

**Disturbance and clone size associations**

We investigated the association between the spatial distribution of large clones and the distance to a well characterized source of disturbance; seagrass burial caused by the highly dynamic sand barrier islands separating the system to the ocean [1]. The large spatial extent of our sampling design resulted in a low density of sampling plots, with an average distance between neighboring plots of 96 m. This unusual sampling design led us to estimate a statistic that would indicate at each sampling plot the probability of finding a clone that was present in the sample *n* or more times. Because four sample units were sampled in each plot, we asked for each of the four multi-locus genotypes in a plot if they had been found five or more times in the whole sample. Finding a MLG that was present in the whole sample five or more times ensured that at least one clonemate would have been found in a different plot (on average at least 96 m apart). A binomial response was produced for the above condition and averaged for each of the four sample units in a plot. We also produced similar estimates for sample units observed 10 and 15, or more times. We named these *clonal probabilities*, *Pc*_5_, *Pc*_10_ and *Pc*_15_, respectively. If larger clones are indeed found away from the disturbance source, plots sampled in such areas should on average have higher *Pc*. We binned the average plot *Pc* in distance classes away from the disturbance source. We did this by estimating the shortest straight distance from the sampling plot to a shape object contouring the sand barrier island. We also fitted a linear regression of *Pc* on distance from the barrier island. A permutation test was used to test the null hypothesis that *Pc* was not associated to the distance from the barrier island. Null distributions were obtained by repeating 1 000 times a permutation of sampling coordinates and recalculating each time the *Pc* mean values per distance class and the regression slope. A particular observed *Pc* mean value, or regression slope of mean PC on distance from barrier island, rejected the null hypothesis if smaller or larger than the 2.5% and 97.5% percentiles, respectively, of the permuted null distributions.

**References**

1. Cunha AH, Santos RP, Gaspar AP, Bairros MF. Seagrass landscape-scale changes in response to disturbance created by the dynamics of barrier-islands: A case study from Ria Formosa (Southern Portugal). 2005;64: 636–644.
